# Oculoplastics and Augmented Intelligence: A Literature Review

**DOI:** 10.3390/jcm14196875

**Published:** 2025-09-28

**Authors:** Edsel Ing, Mostafa Bondok

**Affiliations:** 1 Department of Ophthalmology and Visual Sciences, University of Alberta, Edmonton, AB T6G 2R3, Canada; 2 Department of Ophthalmology and Vision Sciences, University of Toronto Temerty School of Medicine, Toronto, ON M5S 2L9, Canada; 3 Section of Ophthalmology, Department of Surgery, Cumming School of Medicine, University of Calgary, Calgary, AB T2N 4N1, Canada

**Keywords:** artificial intelligence, augmented intelligence, large language models, machine learning, oculoplastics, ptosis, eyelid neoplasms, thyroid eye disease, orbital fractures, giant cell arteritis

## Abstract

Artificial intelligence (AI) and augmented intelligence have significant potential in oculoplastics, offering tools for diagnosis, treatment recommendations, and administrative efficiency. This article discusses current and potential applications of AI in ptosis, eyelid and conjunctival cancer, thyroid-associated orbitopathy (TAO), giant cell arteritis (GCA), and orbital fractures. AI-based programs can assist in screening, predicting surgical outcomes, and improving patient care through data-driven decisions. Privacy concerns, particularly with the use of facial and ocular photographs, require robust solutions, including blockchain, federated learning and steganography. Large generalizable datasets with adequate validation are crucial for future AI development. While AI can assist in clinical decision-making and administrative tasks, physician oversight remains critical to prevent potential errors. Large language models like ChatGPT also have the potential to counsel patients, although further validation is needed to ensure accuracy and patient safety. Ultimately, AI should be regarded as an augmentative tool that supports, rather than replaces, physician expertise in oculoplastic care.

## 1. Introduction

While artificial intelligence (AI) can operate autonomously, augmented intelligence refers to the use of AI-generated insights to support and enhance human decision-making [1]. In clinical practice, augmented intelligence is generally preferred, as physician oversight remains essential for patient safety and diagnostic accuracy [1].

In oculoplastic surgery, AI has demonstrated potential in diagnosing, quantifying, classifying, predicting outcomes, and generating treatment recommendations for conditions such as ptosis, eyelid and conjunctival malignancies, thyroid-associated orbitopathy (thyroid eye disease), orbital fractures, and giant cell arteritis (GCA). These systems can process diverse datasets and sources, including electronic medical records (EMRs), blood test results, medical imaging, facial images, genetic tests, clinical trials, and monitoring devices to provide actionable clinical insights [2].

Large language learning models (LLMs) have been shown to be effective in the generation of oculoplastic differential diagnosis [3,4], and in counseling patients with oculoplastics diseases [5]. Beyond direct clinical applications, AI can streamline practice management by automating scheduling, pre-populating charts, reviewing EMR data, securing insurance approvals, coding and billing, and enhancing safety through prescription cross-checks and preoperative safety checks [6].

As AI applications in oculoplastics rapidly evolve, the development of large, representative datasets and validated algorithms remains critical. Training datasets must be accurately annotated by domain experts and reflect the demographics of the affected patient populations to avoid perpetuating racial, ethnic, or sociodemographic inequities. Privacy concerns, particularly with facial and ocular photographs, must also be carefully managed. In this review, we summarize current applications of AI in oculoplastics and highlight opportunities for future development.

## 2. Methodology

A literature search of Ovid MEDLINE, Ovid Embase, and the Cochrane Central Register of Controlled Trials (CENTRAL) was performed from database inception to 1 January 2025 for this narrative review. The search strategy was developed in consultation with an experienced biomedical sciences librarian, and the full Ovid MEDLINE strategy is provided in Supplemental Table S1. Because this study was based exclusively on publicly available data, it was deemed exempt from ethics review by the University of Alberta Health Research Ethics Board (HREB).

Studies were eligible for inclusion if they examined the use of artificial intelligence (AI) in oculoplastics. All branches of AI (e.g., rule-based algorithms, machine learning, deep learning, large language models) and all application domains (e.g., outcome prediction, screening and risk stratification, task automation, decision support, and treatment planning) were considered. Both observational and experimental studies involving human participants were included. The exclusion criteria were as follows: (1) studies not published in English; (2) non-human studies; (3) studies primarily focused on non-oculoplastic diseases; (4) systematic reviews, commentaries, conference abstracts, editorials, and other non–peer-reviewed publications. Selection of higher quality, relevant studies was conducted descriptively, and articles were organized by topic, relevance, and conclusions through a collaborative, iterative process.

## 3. Ptosis

Early studies have utilized deep learning to identify the presence of ptosis or blepharoptosis using facial images [7,8]. Hung et al. assessed the performance of several convolutional neural networks (CNN) on their ability to identify blepharoptosis from clinical photographs, with the best model achieving a sensitivity of 90.1%, even without pre-training [8]. More advanced programs have expanded this capability, assessing key parameters such as margin-reflex distances (MRD-1, MRD-2), levator function, brow height, and dermatochalasis [9,10,11,12,13], potentially streamlining referrals and prepopulating EMRs. To enhance accessibility, AI-based smartphone applications have also been validated [14,15]. Chen et al. utilized the Medical AI assistant (MAIA) to predict MRD1, MRD2 and LF from photographs in primary, upgaze, and downgaze positions. Their results demonstrated excellent correlation for MRD1 (r = 0.91) and MRD2 (r = 0.88) with gold-standard measurements, though levator function showed only moderate correlation (r = 0.73) [14]. Similarly, Tabuchi et al. re-trained MobileNetV2 and achieved 83.0% sensitivity and 82.5% specificity for detecting blepharoptosis from tablet-based photographs [15].

Some AI programs can predict which ptosis surgical approach is most optimal for a patient based on two- (2D) and three-dimensional (3D) ocular parameters, demonstrating high predictive accuracy [16]. DALL-E is a text-to-image artificial intelligence model developed by OpenAI. It takes written prompts and generates novel images based on the description. AI image generators like DALL-E that can even be used to predict the possible post-operative appearance of patients with ptosis [17]. Sun et al. developed their own deep learning-based models to demonstrate to patients’ postoperative appearance after blepharoptosis surgery, with a high degree of accuracy and patient-rated satisfaction [18]. In clinical practice, these technologies may help patients better understand expected surgical outcomes, reduce anxiety, and facilitate realistic expectations. Their utility could be further enhanced if predictions were tailored to a surgeon’s individual techniques and prior outcomes.

Due to differences in photograph sizes, face turns, chin position, direction of gaze, and dynamic factors of the face (e.g., facial expressions, brow movements), objective comparisons of pre- and post-operative images after eyelid surgery can be difficult. Bahçeci Şimşek and Sirolu developed a computer vision algorithm using a facial landmarking system to normalize and calibrate these photos for improved objective comparisons [19]. However, variables like blinking, make-up, dermatochalasis, brow ptosis, frontalis overaction, hypotropia and lid retraction may confound the interpretation of static ptosis measurements. In addition, mobility and animation effects, including brow activation, frontalis recruitment, gaze changes, room lighting, and the timing of photo capture introduce variability that may depend on how the image is obtained and prepared, further impacting AI accuracy. The use of static photos may hamper the diagnosis of myasthenia gravis, essential blepharospasm, hemifacial spasm and compressive third nerve palsy with synkinesis. Future AI systems that automatically analyze both dynamic and static images with lid height, anisocoria, iris heterochromia, pupil gaze synkinesis, eye movements, lid gaze synkinesis, fornix examination, fundus images, enophthalmos and orbital retropulsion would improve the diagnosis of ptosis associated with neuro-ophthalmic and orbital conditions.

## 4. Eyelid and Conjunctival Cancer

AI programs are being developed to predict the risk of eyelid neoplasms from photographs. In a study of 1417 images from three hospitals, a CNN-based deep learning classification system demonstrated performance comparable to ophthalmologists in the diagnosis of eyelid malignant melanoma (MM) [20], though the absence of sociodemographic data limits the generalizability of the results across diverse racial, ethnic, and other underrepresented patient populations. Further validation is also needed to ensure non-inferiority to oculoplastic specialists. For the diagnosis of skin malignancies in general, few algorithms have been approved for clinical use. MelaFind, computer-vision system, was the first dermatology AI device to gain FDA approval and has shown impressive diagnostic capabilities for cutaneous melanomas compared to dermatologists [21]. Notably, only the lesions scheduled for biopsy in toto were evaluated in this trial, making benign lesions identified in this study not representative of benign lesions in the general population that would otherwise not be biopsied. This may limit the generalizability of specificity in the clinical setting. Similar systems developed specifically for eyelid lesions may aid in oculoplastics triage. The type of periocular skin cancer being diagnosed by AI should be considered when assessing performance; non-AI studies have found that basal cell carcinomas and chalazia are more easily diagnosed by oculoplastics specialists compared to squamous cell carcinoma and possibly melanoma or sebaceous carcinoma [22]. The inclusion of the patient’s age, history of sun exposure, skin cancer elsewhere, and other risk factors would also increase accuracy and quantify the sociodemographic diversity of the training dataset. The expert annotation of large training databases with uniform photographic exposure composed of patients from diverse races, ethnicities, and different levels of skin pigmentation is ideal.

Beyond clinical diagnostics, Tan et al., demonstrated the utility of a decision-tree model for assessing the surgical complexity of periocular basal cell carcinoma (pBCC) excisions [23]. However, this was done with a relatively small sample size at two centres in New Zealand, and replication of these results at different centers would improve generalizability. Ideally, the training photos should be of uniform high quality to show lash loss, ulceration, infiltration, telangiectasia, and be accompanied by lid eversion. In ocular pathology, Jiang et al. introduced a self-supervised learning (SSL) framework that outperforms traditional supervised models in diagnosing eyelid malignant melanoma (MM) from whole-slide pathological images [24]. These results, however, were not externally validated using a clinical dataset. Similarly, Wang et al. reported a diagnostic accuracy of 94.9% using a pathologist-labeled deep learning system on H&E-stained whole-slide images [25]. The study was however limited by a small sample size and contained overlap in patients used in the train/validation/test sets. With the growing demand for ocular pathologists [26], future research should further explore the potential of AI in enhancing the accuracy and efficiency of pathological diagnoses for eyelid lesions.

## 5. Thyroid-Associated Orbitopathy

The ability to predict which patients will develop moderate to severe thyroid eye disease, more appropriately termed thyroid-associated orbitopathy (TAO), is an important asset as the therapeutic options for TAO have progressed well beyond selenium, glucocorticoids, radiation and surgery [27]. Several studies have demonstrated the ability to detect and grade TAO using eye morphology from facial photographs [28,29,30,31,32,33]. Yan et al. analyzed slit-lamp and facial images from 156 patients with a deep learning model trained via supervised learning to predict Clinical Activity Scores (CAS), achieving an accuracy of 0.91 and specificity of 0.83 for diagnosing active disease [33]. Similarly, Shin et al. used smartphone photographs alone in a machine learning system to assess CASs, reporting a specificity of 0.85 [28], while Moon et al. reported a specificity of 0.83 [31]. Future studies should evaluate the predictive power of these models over time, to confirm their clinical utility and robustness.

Additionally, AI applications using deep learning on computed tomography (CT) images show promise in measuring exophthalmos [34], quantifying extraocular muscle (EOM) enlargement [35,36], and performing volumetric analysis of orbital fat [36]. Lai et al. further demonstrated the utility of dynamic regional homogeneity (dReHo) alterations combined with support vector machine (SVM) classification to differentiate TAO patients from healthy controls, achieving an accuracy of 0.66–0.69 [37]. AI-based quantitative evaluation of retinal vascular parameters has also been explored as a potential diagnostic tool for TAO [38], but further research is needed to demonstrate the extent of its clinic utility.

For patients already diagnosed with TAO, CNNs have been used to assess disease activity through static radiologic images [39,40,41,42,43]. For example, Lin et al. trained a CNN on MRI images to detect active-phase disease, achieving a specificity of 0.86 and sensitivity of 0.89 [40], while Li et al. obtained good, but slightly less favorable results using contrast-enhanced MRI [43]. Lee et al. developed a neural network-based method using orbital CT scans, reporting a specificity of 0.79 and sensitivity of 0.78 [41]. Yao et al. combined CT and SPECT/CT images in their model, achieving a specificity of 0.84 and sensitivity of 0.85 [39]. These differences likely reflect variations in algorithm design, input data, and imaging modality, rather than indicating that one modality is inherently superior. CT has advantages for anatomical assessment except at the orbital apex, while MRI better characterizes inflammatory activity. Beyond diagnosis, AI prediction models can also help identify patients unlikely to benefit from intravenous glucocorticoid therapy, thus expediting appropriate treatment [44]. The XGBoost model, for example, predicted steroid treatment response with 86% accuracy by combining clinical and laboratory data such as thyroid-stimulating hormone (TSH), thyroid-stimulating immunoglobulin (TSI), and low-density lipoprotein (LDL) levels, as well as EOM limitation [45]. Other studies have demonstrated that T2-weighted Magnetic resonance imaging (MRI)-derived radiomics offer a promising non-invasive approach that uses machine learning to predict pretreatment response to glucocorticoids TAO patients [44,46,47,48].

An ideal AI tool for TAO would integrate the timing and progression of photographic images, exophthalmometry, CT/MRI data, lab results, with smoking history, family history, and genetic markers. It should accurately calculate an inflammatory activity score (e.g., EUGOGO CAS or ITEDS score). Remote monitoring of calibrated photos could facilitate continuous assessment between visits, detecting changes that may require intervention.

Using demographic, clinical and genetic data AI-driven precision medicine prediction models for TAO will incorporate the side-effect profiles and costs of immunosuppressants like IGF1 inhibitors (e.g., teprotumumab), IL-6 inhibitors (e.g., tocilizumab), and anti-FcRn receptors for precision medicine. Ultimately, the algorithm would recommend the most effective immunosuppressant or combination therapies and the need and timing for emergent surgery, or elective surgery in the quiescent phase.

## 6. Giant Cell Arteritis

In North America, oculoplastic surgeons are frequently asked to perform temporal artery biopsy in patients with suspected giant cell arteritis (GCA). A ten factor, TRIPOD-validated, shallow neural network prediction model for GCA is freely available online [49], and may aid in diagnostic decision-making, the need for glucocorticoid treatment, and decrease the need for temporal artery biopsy in patients at low risk for GCA.

In Europe, ultrasound is frequently used as first line test for GCA, though it has a false positive rate of up to 5% [50]. Combining ultrasound and AI-driven diagnostic algorithms to improve the early diagnosis and treatment of GCA has the potential for AI to revolutionize the diagnostic landscape by providing faster, less invasive results [51]. Color Doppler ultrasound (CDU) of the temporal arteries is a non-invasive tool that has shown promise, though its diagnostic role in GCA is still being defined [52]. A significant limitation of CDU is the poor inter-operator reproductivity. A retrospective study of CDU images used a semantic segmentation technique using a U-Net CNN for identifying the halo sign on CDU images with a 95% specificity and 60% sensitivity [52]. Another study using a backpropagation neural network on the vasculitis database of the American College of Rheumatology achieved a specificity of 91.9% and sensitivity of 94.4% when classifying true GCA cases compared to other vasculitis cases [53]. Algorithms that combine prediction models with ultrasound, MRI, or future genetic markers may further reduce the burden of temporal artery biopsies. A limitation of current AI approaches is that variability in ultrasound acquisition across centres can reduce algorithm accuracy, as demonstrated in studies where automated analysis of CDU images performed inconsistently between sites. Adherence to standardized imaging protocols, such as the 2018 EULAR recommendations for large-vessel vasculitis, could reduce image quality bias and improve the generalizability of AI models while minimizing the amount of training data required.

Determining the optimal dose and duration of glucocorticoid therapy and/or tocilizmuab in GCA can be challenging. A study comparing machine learning models found that random forest (sensitivity = 60.0%, specificity = 77.6%) and logistic regression (sensitivity = 62.5%, specificity = 74.6%) models outperformed the decision tree (sensitivity = 47.5%, specificity = 70.2%) model in predicting GCA relapse after glucocorticoid tapering [54]. Future AI applications may help optimize glucocorticoid and tocilizumab dosing in patients with GCA based on clinical signs, symptoms, ultrasound and serum markers and reduce medication side effects and cost [51].

## 7. Orbital Fractures

AI applications that use deep learning models to detect midfacial fractures on facial bone CT images show promise in emergency and primary care settings [55]. A review of blowout fracture area measurements concluded that automated methods, such as computer-aided measurements and CT-based volumetric analysis, offer greater accuracy and reliability compared to manual and semiautomatic techniques [56]. Deep learning-based automatic segmentation of CT images for orbital fractures may enable faster and more cost-effective creation of reference 3D anatomical models for surgeons [57] and assist in the 3D printing fracture implants. By combining facial photos, history and imaging findings, AI has the potential to determine which acute fractures have associated globe rupture, are at risk of orbital compartment syndrome or unresolved diplopia, detect isodense foreign bodies, and predict objectionable enophthalmos. AI integrated into EMRs may potentially flag victims of child abuse or intimate partner violence.

## 8. Administrative Tasks and Patient Counseling

The integration of AI into oculoplastics and an enterprise system may help decrease the burden of administrative tasks such as scribing, physician orders, in-basket message responses, chart summaries, coding, billing, form completion, insurance authorization, scheduling, supply chains and inventory and patient flow [6]. By saving time on these tasks, AI allows physicians to spend more time with patients, improving both patient and physician well-being. In some cases, replies to patient queries from chatbots were rated as more empathetic than responses from physicians [58]. However, if AI systems are inaccurate, the time and effort required to correct administrative mistakes can be substantial and pose additional risks to the patient [59].

Large language models (LLMs) like ChatGPT have potential applications in patient counseling. In the context of oculoplastic conditions, Rajabi et al., showed that the responses ChatGPT 3.5 offered to patients with TAO for commonly asked questions were consistent with established guidelines, and rated as sufficiently detailed and comprehensible without excessive medical jargon [60]. Another study using an unspecified version of ChatGPT for blepharoptosis pre-surgical counseling found chatbot responses to be effective, though responses were rated more poorly by oculoplastic surgeons compared to non-physician evaluators [61]. A study on the ability of ChatGPT 3.5 to answer 11 common questions on blepharoptosis found responses to be only 61.3% accurate [62], and thus should be used cautiously as an adjust tool that should not replace advice from medical professionals. In another study, ChatGPT 4.0 was asked one hundred questions across 10 categories of oculofacial disease including proptosis, thyroid-associated orbitopathy, lid malpositions, and dacryocystitis, providing mostly appropriate responses when evaluated by seven board-certified oculoplastic surgeons [5].

The future of AI in health care requires the integration of the classic Lean Six Sigma DMAIC principles (Define, Measure, Analyze, Improve, Control) of business and product manufacturing into a DDMLAICE (Define, Data, Machine Learning, Artificial Intelligence, Control, Evaluate) paradigms [63]. Through continuous learning and adaptation, AI systems can be developed to deliver accurate, reliable, consistent and sustainable results with minimal waste.

## 9. Privacy Concerns

Privacy concerns are particularly significant when dealing with facial and ocular photographic data. Techniques such as steganography can mask patient data [64], while watermarking and blockchain technology can enhance data exchange security [65]. When multiple sites are participating in research or clinical care, federated learning can be applied [66]. With federated machine learning models, the data is not pooled for analysis at a central location [66]. Instead of data sharing, the algorithm moves to the different sites to train on the individual datasets. This approach mitigates the risk of large-scale data breaches by creating a shared global model without transferring sensitive data to a single location.

## 10. Conclusions & Future Directions

AI is poised to become more personalized, predictive, preventive, and participatory. As it evolves, AI will integrate dynamic images, wearable devices, and diverse data sources to provide real-time insights into both individual patient care and broader public health trends. This will allow for the interpretation of dynamic images and videos, and the combination of clinical data, genetic information, and environmental factors to monitor both patient health and epidemiological patterns to monitor individual patient health, community health, and epidemiological trends [2]. Furthermore, AI carries the risk of exacerbating existing inequities. Selection bias from non-representative training and validation datasets, as well as measurement bias from unmeasured confounders, can compromise an AI model’s ability to accurately infer relationships. Ensuring equitable AI implementation requires both diverse datasets and rigorous evaluation of model performance across demographic subgroups [67]. A critical first step is the systematic collection and reporting of sociodemographic characteristics to ensure representative study samples. Unfortunately, most studies in this review have not followed this practice. As our search was limited to core medical databases, studies published in engineering-focused or interdisciplinary databases may not have been captured.

In summary, AI may assist in the triage, diagnosis, documentation, and management of oculoplastic diseases. Large language models (LLMs) can also assist in answering patient questions. However, while large, generalizable datasets are needed, privacy concerns remain a challenge due to the identifiable nature of facial and ocular data. Continued validation of AI instruments is required. Diagnostic prediction models should adhere to the updated 2024 Transparent Reporting of a multivariable prediction model for Individual Prognosis or Diagnosis + AI (TRIPOD-AI) guidelines. Though LLMs have the potential to provide accurate and empathetic responses to many patient queries, physician oversight is needed to address potential errors. LLMs may hallucinate or produce factually incorrect/nonsensical outputs [68], especially in response to complex questions. The convergence of AI, static and dynamic digital images, large data sets and genetic markers holds great promise in the future of oculoplastics and medicine, but human oversight of AI remains imperative. AI should not make autonomous clinical decisions but serve as a tool that can augment physician decision-making.

## Data Availability

The original contributions presented in the study are included in the article, further inquiries can be directed to the corresponding author.

## Supplementary Materials

The following supporting information can be downloaded at: https://www.mdpi.com/article/10.3390/jcm14196875/s1, Table S1: Ovid MEDLINE Search Strategy [Ovid MEDLINE(R) ALL 1946 to 1 January 2025].

## References

[1] Bazoukis G., Hall J., Loscalzo J., Antman E.M., Fuster V., Armoundas A.A. (2022). The inclusion of augmented intelligence in medicine: A framework for successful implementation. Cell Rep. Med..

[2] Rajpurkar P., Chen E., Banerjee O., Topol E.J. (2022). AI in health and medicine. Nat. Med..

[3] Balas M., Ing E.B. (2023). Conversational AI Models for ophthalmic diagnosis: Comparison of ChatGPT and the Isabel Pro Differential Diagnosis Generator. JFO Open Ophthalmol..

[4] Ing E.B., Balas M., Nassrallah G., DeAngelis D., Nijhawan N. (2023). The Isabel Differential Diagnosis Generator for Orbital Diagnosis. Ophthalmic Plast. Reconstr. Surg..

[5] Balas M., Janic A., Daigle P., Nijhawan N., Hussain A., Gill H., Lahaie G.L., Belliveau M.J., Crawford S.A., Arjmand P. (2024). Evaluating ChatGPT on Orbital and Oculofacial Disorders: Accuracy and Readability Insights. Ophthalmic Plast. Reconstr. Surg..

[6] Yin J., Ngiam K.Y., Teo H.H. (2021). Role of Artificial Intelligence Applications in Real-Life Clinical Practice: Systematic Review. J. Med. Internet Res..

[7] Abascal Azanza C., Barrio-Barrio J., Ramos Cejudo J., Ybarra Arrospide B., Devoto M.H. (2023). Development and validation of a convolutional neural network to identify blepharoptosis. Sci. Rep..

[8] Hung J.Y., Perera C., Chen K.W., Myung D., Chiu H.K., Fuh C.S., Hsu C.R., Liao S.L., Kossler A.L. (2021). A deep learning approach to identify blepharoptosis by convolutional neural networks. Int. J. Med. Inform..

[9] Lou L., Cao J., Wang Y., Gao Z., Jin K., Xu Z., Zhang Q., Huang X., Ye J. (2021). Deep learning-based image analysis for automated measurement of eyelid morphology before and after blepharoptosis surgery. Ann. Med..

[10] Lou L., Yang L., Ye X., Zhu Y., Wang S., Sun L., Qian D., Ye J. (2019). A Novel Approach for Automated Eyelid Measurements in Blepharoptosis Using Digital Image Analysis. Curr. Eye Res..

[11] Nam Y., Song T., Lee J., Lee J.K. (2024). Development of a neural network-based automated eyelid measurement system. Sci. Rep..

[12] Schulz C.B., Clarke H., Makuloluwe S., Thomas P.B., Kang S. (2023). Automated extraction of clinical measures from videos of oculofacial disorders using machine learning: Feasibility, validity and reliability. Eye.

[13] Van Brummen A., Owen J.P., Spaide T., Froines C., Lu R., Lacy M., Blazes M., Li E., Lee C.S., Lee A.Y. (2021). PeriorbitAI: Artificial Intelligence Automation of Eyelid and Periorbital Measurements. Am. J. Ophthalmol..

[14] Chen H.C., Tzeng S.S., Hsiao Y.C., Chen R.F., Hung E.C., Lee O.K. (2021). Smartphone-Based Artificial Intelligence-Assisted Prediction for Eyelid Measurements: Algorithm Development and Observational Validation Study. JMIR mHealth uHealth.

[15] Tabuchi H., Nagasato D., Masumoto H., Tanabe M., Ishitobi N., Ochi H., Shimizu Y., Kiuchi Y. (2022). Developing an iOS application that uses machine learning for the automated diagnosis of blepharoptosis. Graefes Arch. Clin. Exp. Ophthalmol..

[16] Song X., Tong W., Lei C., Huang J., Fan X., Zhai G., Zhou H. (2021). A clinical decision model based on machine learning for ptosis. BMC Ophthalmol..

[17] Balas M., Micieli J.A., Wulc A., Ing E.B. (2024). Text-to-image artificial intelligence models for preoperative counselling in oculoplastics. Can. J. Ophthalmol..

[18] Sun Y., Huang X., Zhang Q., Lee S.Y., Wang Y., Jin K., Lou L., Ye J. (2022). A Fully Automatic Postoperative Appearance Prediction System for Blepharoptosis Surgery with Image-based Deep Learning. Ophthalmol. Sci..

[19] Bahceci Simsek I., Sirolu C. (2021). Analysis of surgical outcome after upper eyelid surgery by computer vision algorithm using face and facial landmark detection. Graefes Arch. Clin. Exp. Ophthalmol..

[20] Li Z., Qiang W., Chen H., Pei M., Yu X., Wang L., Li Z., Xie W., Wu X., Jiang J. (2022). Artificial intelligence to detect malignant eyelid tumors from photographic images. NPJ Digit. Med..

[21] Monheit G., Cognetta A.B., Ferris L., Rabinovitz H., Gross K., Martini M., Grichnik J.M., Mihm M., Prieto V.G., Googe P. (2011). The performance of MelaFind: A prospective multicenter study. Arch. Dermatol..

[22] Hillson T.R., Harvey J.T., Hurwitz J.J., Liu E., Oestreicher J.H., Pashby R.C. (1998). Sensitivity and specificity of the diagnosis of periocular lesions by oculoplastic surgeons. Can. J. Ophthalmol..

[23] Tan E., Lin F., Sheck L., Salmon P., Ng S. (2017). A practical decision-tree model to predict complexity of reconstructive surgery after periocular basal cell carcinoma excision. J. Eur. Acad. Dermatol. Venereol..

[24] Jiang Z., Wang L., Wang Y., Jia G., Zeng G., Wang J., Li Y., Chen D., Qian G., Jin Q. (2024). A Self-Supervised Learning Based Framework for Eyelid Malignant Melanoma Diagnosis in Whole Slide Images. IEEE/ACM Trans. Comput. Biol. Bioinform..

[25] Wang L., Ding L., Liu Z., Sun L., Chen L., Jia R., Dai X., Cao J., Ye J. (2020). Automated identification of malignancy in whole-slide pathological images: Identification of eyelid malignant melanoma in gigapixel pathological slides using deep learning. Br. J. Ophthalmol..

[26] Stalhammar G., Lardner E., Georgsson M., Seregard S. (2023). Increasing demand for ophthalmic pathology: Time trends in a laboratory with nationwide coverage. BMC Ophthalmol..

[27] Patel A., Yang H., Douglas R.S. (2019). A New Era in the Treatment of Thyroid Eye Disease. Am. J. Ophthalmol..

[28] Shin K., Choung H., Lee M.J., Kim J., Lee G.M., Kim S., Kim J.H., Oh R., Park J., Lee S.M. (2024). A Preliminary Evaluation of the Diagnostic Performance of a Smartphone-Based Machine Learning-Assisted System for Evaluation of Clinical Activity Score in Digital Images of Thyroid-Associated Orbitopathy. Thyroid.

[29] Huang X., Ju L., Li J., He L., Tong F., Liu S., Li P., Zhang Y., Wang X., Yang Z. (2022). An Intelligent Diagnostic System for Thyroid-Associated Ophthalmopathy Based on Facial Images. Front. Med..

[30] Karlin J., Gai L., LaPierre N., Danesh K., Farajzadeh J., Palileo B., Taraszka K., Zheng J., Wang W., Eskin E. (2023). Ensemble neural network model for detecting thyroid eye disease using external photographs. Br. J. Ophthalmol..

[31] Moon J.H., Shin K., Lee G.M., Park J., Lee M.J., Choung H., Kim N. (2022). Machine learning-assisted system using digital facial images to predict the clinical activity score in thyroid-associated orbitopathy. Sci. Rep..

[32] Shao J., Huang X., Gao T., Cao J., Wang Y., Zhang Q., Lou L., Ye J. (2023). Deep learning-based image analysis of eyelid morphology in thyroid-associated ophthalmopathy. Quant. Imaging Med. Surg..

[33] Yan C., Zhang Z., Zhang G., Liu H., Zhang R., Liu G., Rao J., Yang W., Sun B. (2024). An ensemble deep learning diagnostic system for determining Clinical Activity Scores in thyroid-associated ophthalmopathy: Integrating multi-view multimodal images from anterior segment slit-lamp photographs and facial images. Front. Endocrinol..

[34] Zhang Y., Rao J., Wu X., Zhou Y., Liu G., Zhang H. (2023). Automatic measurement of exophthalmos based orbital CT images using deep learning. Front. Cell Dev. Biol..

[35] Hanai K., Tabuchi H., Nagasato D., Tanabe M., Masumoto H., Miya S., Nishio N., Nakamura H., Hashimoto M. (2022). Automated detection of enlarged extraocular muscle in Graves’ ophthalmopathy with computed tomography and deep neural network. Sci. Rep..

[36] Alkhadrawi A.M., Lin L.Y., Langarica S.A., Kim K., Ha S.K., Lee N.G., Do S. (2024). Deep-Learning Based Automated Segmentation and Quantitative Volumetric Analysis of Orbital Muscle and Fat for Diagnosis of Thyroid Eye Disease. Investig. Ophthalmol. Vis. Sci..

[37] Lai P.H., Hu R.Y., Huang X. (2024). Alterations in dynamic regional homogeneity within default mode network in patients with thyroid-associated ophthalmopathy. Neuroreport.

[38] Jiang X., Dong L., Luo L., Zhou D., Ling S., Li D. (2024). Artificial Intelligence-based quantitative evaluation of retinal vascular parameters in thyroid-associated ophthalmopathy. Endocrine.

[39] Yao N., Li L., Gao Z., Zhao C., Li Y., Han C., Nan J., Zhu Z., Xiao Y., Zhu F. (2023). Deep learning-based diagnosis of disease activity in patients with Graves’ orbitopathy using orbital SPECT/CT. Eur. J. Nucl. Med. Mol. Imaging.

[40] Lin C., Song X., Li L., Li Y., Jiang M., Sun R., Zhou H., Fan X. (2021). Detection of active and inactive phases of thyroid-associated ophthalmopathy using deep convolutional neural network. BMC Ophthalmol..

[41] Lee J., Lee S., Lee W.J., Moon N.J., Lee J.K. (2023). Neural network application for assessing thyroid-associated orbitopathy activity using orbital computed tomography. Sci. Rep..

[42] Wang M., Li G., Dong L., Hou Z., Zhang J., Li D. (2024). Severity Identification of Graves Orbitopathy via Random Forest Algorithm. Horm. Metab. Res..

[43] Li Y., Ma J., Xiao J., Wang Y., He W. (2024). Use of extreme gradient boosting, light gradient boosting machine, and deep neural networks to evaluate the activity stage of extraocular muscles in thyroid-associated ophthalmopathy. Graefes Arch. Clin. Exp. Ophthalmol..

[44] Wang Y., Wang H., Li L., Li Y., Sun J., Song X., Zhou H. (2021). Novel observational study protocol to develop a prediction model that identifies patients with Graves’ ophthalmopathy insensitive to intravenous glucocorticoids pulse therapy. BMJ Open.

[45] Park J., Kim J., Ryu D., Choi H.Y. (2023). Factors related to steroid treatment responsiveness in thyroid eye disease patients and application of SHAP for feature analysis with XGBoost. Front. Endocrinol..

[46] Hu H., Chen L., Zhang J.L., Chen W., Chen H.H., Liu H., Shi H.B., Wu F.Y., Xu X.Q. (2022). T_2_-Weighted MR Imaging-Derived Radiomics for Pretreatment Determination of Therapeutic Response to Glucocorticoid in Patients With Thyroid-Associated Ophthalmopathy: Comparison With Semiquantitative Evaluation. J. Magn. Reson. Imaging.

[47] Zhang H., Jiang M., Chan H.C., Zhang H., Xu J., Liu Y., Zhu L., Tao X., Xia D., Zhou L. (2024). Whole-orbit radiomics: Machine learning-based multi- and fused- region radiomics signatures for intravenous glucocorticoid response prediction in thyroid eye disease. J. Transl. Med..

[48] Wang Y.Y., Wu Q., Chen L., Chen W., Yang T., Xu X.Q., Wu F.Y., Hu H., Chen H.H. (2021). Texture analysis of orbital magnetic resonance imaging for monitoring and predicting treatment response to glucocorticoids in patients with thyroid-associated ophthalmopathy. Endocr. Connect..

[49] Ing E.B., Miller N.R., Nguyen A., Su W., Bursztyn L., Poole M., Kansal V., Toren A., Albreki D., Mouhanna J.G. (2019). Neural network and logistic regression diagnostic prediction models for giant cell arteritis: Development and validation. Clin. Ophthalmol..

[50] Fernandez-Fernandez E., Monjo-Henry I., Bonilla G., Plasencia C., Miranda-Carus M.E., Balsa A., De Miguel E. (2020). False positives in the ultrasound diagnosis of giant cell arteritis: Some diseases can also show the halo sign. Rheumatology.

[51] Avasarala J., Das S., Keshavamurthy S. (2024). Point-of-Care Ultrasound With Artificial Intelligence-Driven Diagnostics in Giant Cell Arteritis: Blindness Prevention on a Global Scale. J. Rheumatol..

[52] Roncato C., Perez L., Brochet-Guegan A., Allix-Beguec C., Raimbeau A., Gautier G., Agard C., Ploton G., Moisselin S., Lorcerie F. (2020). Colour Doppler ultrasound of temporal arteries for the diagnosis of giant cell arteritis: A multicentre deep learning study. Clin. Exp. Rheumatol..

[53] Astion M.L., Wener M.H., Thomas R.G., Hunder G.G., Bloch D.A. (1994). Application of neural networks to the classification of giant cell arteritis. Arthritis Rheum..

[54] Venerito V., Emmi G., Cantarini L., Leccese P., Fornaro M., Fabiani C., Lascaro N., Coladonato L., Mattioli I., Righetti G. (2022). Validity of Machine Learning in Predicting Giant Cell Arteritis Flare After Glucocorticoids Tapering. Front. Immunol..

[55] Morita D., Kawarazaki A., Soufi M., Otake Y., Sato Y., Numajiri T. (2024). Automatic detection of midfacial fractures in facial bone CT images using deep learning-based object detection models. J. Stomatol. Oral. Maxillofac. Surg..

[56] Kang D. (2023). Evaluating the Accuracy and Reliability of Blowout Fracture Area Measurement Methods: A Review and the Potential Role of Artificial Intelligence. J. Craniofac. Surg..

[57] Morita D., Kawarazaki A., Koimizu J., Tsujiko S., Soufi M., Otake Y., Sato Y., Numajiri T. (2023). Automatic orbital segmentation using deep learning-based 2D U-net and accuracy evaluation: A retrospective study. J. Craniomaxillofac. Surg..

[58] Ayers J.W., Poliak A., Dredze M., Leas E.C., Zhu Z., Kelley J.B., Faix D.J., Goodman A.M., Longhurst C.A., Hogarth M. (2023). Comparing Physician and Artificial Intelligence Chatbot Responses to Patient Questions Posted to a Public Social Media Forum. JAMA Intern. Med..

[59] Liu J., Wang C., Liu S. (2023). Utility of ChatGPT in Clinical Practice. J. Med. Internet Res..

[60] Rajabi M.T., Rafizadeh S.M., Ghahvehchian H. (2024). Exploring the Use of ChatGPT in Delivering Evidence-Based Information to Patients with Thyroid Eye Disease. Ophthalmic Plast. Reconstr. Surg..

[61] Shiraishi M., Tanigawa K., Tomioka Y., Miyakuni A., Moriwaki Y., Yang R., Oba J., Okazaki M. (2024). Blepharoptosis Consultation with Artificial Intelligence: Aesthetic Surgery Advice and Counseling from Chat Generative Pre-Trained Transformer (ChatGPT). Aesthetic Plast. Surg..

[62] Shiraishi M., Tomioka Y., Miyakuni A., Ishii S., Hori A., Park H., Ohba J., Okazaki M. (2024). Performance of ChatGPT in Answering Clinical Questions on the Practical Guideline of Blepharoptosis. Aesthetic Plast. Surg..

[63] Sood A.C., Dhull K.S. (2024). The Future of Six Sigma- Integrating AI for Continuous Improvement. Int. J. Innov. Res. Eng. Manag..

[64] Balas M., Rudnisky C., Ing E.B. (2024). Hidden in plain sight: AI-driven steganography and watermarking for secure transmission of ophthalmic data. AJO Int..

[65] Balas M., Wong D.T., Ing E.B. (2024). Blockchain technology: Revolutionizing ophthalmology and patient-centred care. Can. J. Ophthalmol..

[66] Sheller M.J., Edwards B., Reina G.A., Martin J., Pati S., Kotrotsou A., Milchenko M., Xu W., Marcus D., Colen R.R. (2020). Federated learning in medicine: Facilitating multi-institutional collaborations without sharing patient data. Sci. Rep..

[67] Bondok M., Selvakumar R., Asdo A., Naderi B., Zhang C., Wong C., Felfeli T. (2025). Sociodemographic Reporting in Artificial Intelligence Studies of Retinal Diseases: A Critical Appraisal of the Literature. Ophthalmol. Retin..

[68] Chen J.S., Reddy A.J., Al-Sharif E., Shoji M.K., Kalaw F.G.P., Eslani M., Lang P.Z., Arya M., Koretz Z.A., Bolo K.A. (2025). Analysis of ChatGPT Responses to Ophthalmic Cases: Can ChatGPT Think like an Ophthalmologist?. Ophthalmol. Sci..

