# Supplementary material for: Oculoplastics and Augmented Intelligence: A Literature Review
Source: J Clin Med. 2025 Sep 28;14(19):6875. doi: 10.3390/jcm14196875 (PMC12524889; doi:10.3390/jcm14196875)
Supplement: Supplementary file 1 [file jcm-14-06875-s001.zip › jcm-3872341-supplementary.pdf]

Supplemental Material

Table S1. Ovid MEDLINE Search Strategy [Ovid MEDLINE(R) ALL 1946 to January 1, 2025].

Line	Search query
1	artificial intelligence/
2	(artificial adj2 intelligence).mp.
3	machine learning/
4	(machine adj2 learning).mp.
5	deep learning/
6	(deep adj2 learn*).mp.
7	supervised machine learning/
8	(supervised learning OR unsupervised learning).mp.
9	unsupervised machine learning/
10	support vector machine/
11	(support adj2 vector adj2 machine*).mp.
12	neural networks, computer/
13	(computer adj2 neural adj2 network*).mp.
14	neural network*.mp.
15	pattern recognition, automated/
16	(automated adj2 "pattern* recognition").mp.
17	diagnosis, computer-assisted/ or image interpretation, computer-assisted/
18	(("computer-assisted" or "computer assisted") adj2 (diagnos* or "image interpret*")).mp.
19	computational intelligence.mp.
20	bayes theorem/
21	(bayes* adj2 theorem).mp.
22	(naive adj2 bayes).mp.
23	Decision Trees/
24	(decision adj2 tree*).mp.
25	(random adj2 forest*).mp.
26	(boosted adj2 tree*).mp.
27	(reinforcement adj2 learn*).mp.

28	1 or 2 or 3 or 4 or 5 or 6 or 7 or 8 or 9 or 10 or 11 or 12 or 13 or 14 or 15 or 16 or 17 or 18 or 19 or 20 or 21 or 22 or 23 or 24 or 25 or 26 or 27
29	blepharitis/ or blepharitis.mp. or chalazion/ or chalazion.mp. or ectropion/ or ectropion.mp. or entropion/ or entropion.mp. or hordeolum/ or hordeolum.mp. or blepharoptosis/ or (ptosis or blepharoptosis).mp. or eyelid retraction/ or (retraction or lagophthalmos).mp. or trichiasis/ or trichiasis.mp. or distichiasis.mp. or epiblepharon.mp. or dermatochalasis.mp. or brow ptosis.mp. or eyebrow.pt. or (brow adj3 ptosis).mp. or blepharospasm/ or blepharospasm.mp. or hemifacial spasm/ or (hemifacial adj2 spasm).mp.
30	eyelid neoplasms/ or orbital neoplasms/ or (orbital adj (neoplasm* or tumor* or tumour* or malignan* or cancer*)).mp. or lacrimal gland diseases/ or lacrimal gland neoplasms/ or enucleation/ or evisceration/ or exenteration/
31	eyelids/ or eyelid*.mp. or eyelashes/ or eyelash*.mp. or blepharoplasty/ or blepharoplasty.mp. or (brow lift or forehead lift).mp.
32	lacrimal apparatus diseases/ or dacryocystitis/ or dacryocystitis.mp. or dacryostenosis/ or (nasolacrimal adj3 (obstruction or stenosis)).mp. or (epiphora or canaliculitis or canalicul* or punctal stenosis).mp.
33	graves disease/ or graves ophthalmology/ or graves disease.mp. or (orbital tissue or orbital adipose tissue).mp. or enophthalmos.mp.
34	orbital fractures/ or (orbital adj3 (fracture* or blowout or “blow-out”)).mp. or orbital cellulitis/ or orbital cellulitis.mp.
35	29 or 30 or 31 or 32 or 33 or 34
36	28 and 35
36	limit 36 to humans
